# Pharmacological characteristics and efficacy of a novel anti-angiogenic antibody FD006 in corneal neovascularization

**DOI:** 10.1186/1472-6750-14-17

**Published:** 2014-02-27

**Authors:** Qun Wang, Jing Yang, Kun Tang, Longlong Luo, Liqiang Wang, Lei Tian, Yanming Jiang, Jiannan Feng, Yan Li, Beifen Shen, Ming Lv, Yifei Huang

**Affiliations:** 1 Department of Ophthalmology, General Hospital of People’s Liberation Army, No.28 Fuxing Road, Haidian District, Beijing 100853, China; 2 Institute of Basic Medical Sciences, Taiping Road, P.O. Box 130 (3), Beijing 100850, China; 3 Department of Ophthalmology, Aerospace center Hospital, No. 15 Yuquan Road, Haidian District, Beijing 100049, China; 4 Department of Ophthalmology, The second Artillery General Hospital, No.16 Xinwai Road, Xicheng District, Beijing 100088, China

**Keywords:** Neovascularization, Cornea, Bevacizumab, Angiogenesis, Anti-angiogenic treatment

## Abstract

**Background:**

Vascular endothelial growth factor (VEGF) is a key angiogenic factors. It plays an important role in both physiologic and pathologic angiogenesis and increases permeability across the vessels. Using antibody phage display technology, we obtained a novel anti-VEGFA IgG, named as FD006. In this study, the pharmacological characteristics and efficacy of FD006 in corneal neovascularization (CoNV) were evaluated.

**Results:**

FD006 was predicted to have similar binding mode to bevacizumab. Experimental analysis showed that the binding ability of FD006 seemed a little stronger than bevacizumab, for the EC50 of FD006 to bind VEGF analyzed by ELISA was about 0.037 μg/mL while that of bevacizumab was 0.18 μg/mL. Binding kinetics assays showed similar results that FD006 possessed 2-fold higher affinity to bind VEGF than bevacizumab due to slower dissociation rate of FD006; meanwhile, FD006 inhibited the VEGF-induced proliferation of HUVEC with an IC50 value of 0.031 ± 0.0064 μg/ml, which seemed similar or a litter better than bevacizumab (0.047 ± 0.0081 μg/ml). The subconjunctival administration of FD006, bevacizumab or dexamethasone could significantly inhibit the growth of CoNV contrasting to N.S (p < 0.01). At the early stage, FD006 showed better inhibitory effect on the growth of CoNV compared with bevacizumab (p < 0.05). Western blot analysis showed that FD006 could inhibit the expression of VEGF, VEGFR-1, VEGFR-2, MMP-9 and ICAM-1, which could explain its favorable anti-angiogenic activity.

**Conclusions:**

The pharmacological characteristics of FD006 were similar or even a little better than bevacizumab in inhibiting corneal neovascularization.

## Background

Corneal avascularity is necessary for the preservation of corneal transparency and optimal vision [1]. There is a dynamic balance between pro- and anti-angiogenic factors in the cornea that plays a key role in maintaining avascularity. Under normal physiologic conditions, the process of angiogenesis is well controlled, reflecting a perfect balance of endogenous growth factors and suppressors. However, in the condition of persistent hypoxia, chronic inflammation, injury and hereditary stem cell deficiency, the pro-angiogenic growth factors outnumber angiogenesis inhibitors, the balance shifts in favor of angiogenesis, and then the corneal angiogenic privilege could be destroyed [2,3]. Although new blood vessel growth in response to ischemic conditions is to repair the injury, pathologic corneal neovascularization (CoNV) and hyperpermeability can cause decreased vision and failure of transplantation. CoNV is a sight-threatening condition, which is also a challenge for ophthalmologists. The inhibitory effects of steroids, cyclosporine, thalidomide, photodynamictherapy, conjunctival limbal allograft and fine needle diathermy for CoNV vary in clinical and experimental use [4-9]. They are not always effective, sometimes may induce complications.

Angiogenesis, the process by which new vessels sprout from pre-existing vasculature, has become the subject of intense research in recent years and therapies that directly target key factors of neovascular formation are promising. Among the angiogenic factors, vascular endothelial growth factor (VEGF) plays an important role in both physiologic and pathologic angiogenesis and increases permeability across the vessels. Extensive research studies focus on the VEGF families and receptors [10,11]. Given that several studies have demonstrated that the secretion and receptor expression of VEGF were increased during the neovascularization of the cornea, specific molecules have been targeted for drug development and have drawn much attention recently [10-12]. The targeted inhibition of VEGF provides ophthalmologists with an angiogenesis-specific pharmacological approach to treating CoNV and deserves to be evaluated. There are many VEGF inhibitors which are in advanced clinical use for ocular neovascularization, such as Lucentis, Macugen and bevacizumab/Avastin. Administration of these drugs in CoNv has been associated with promising results [13-15].

In our previous work, we screened out a novel anti-VEGF antibody from a natural human scFv phage library which was constructed in our lab. Then, the recombinant vector to express the full antibody was constructed and transfected into mammalian cells. The full IgG, named as FD006, was prepared for further studies. The purpose of the present study was to investigate the pharmacological characteristics and efficacy of FD006 both *in vitro* and *in vivo*.

## Results

### The predicted binding mode of FD006 was similar to bevacizumab

**Figure 1:**
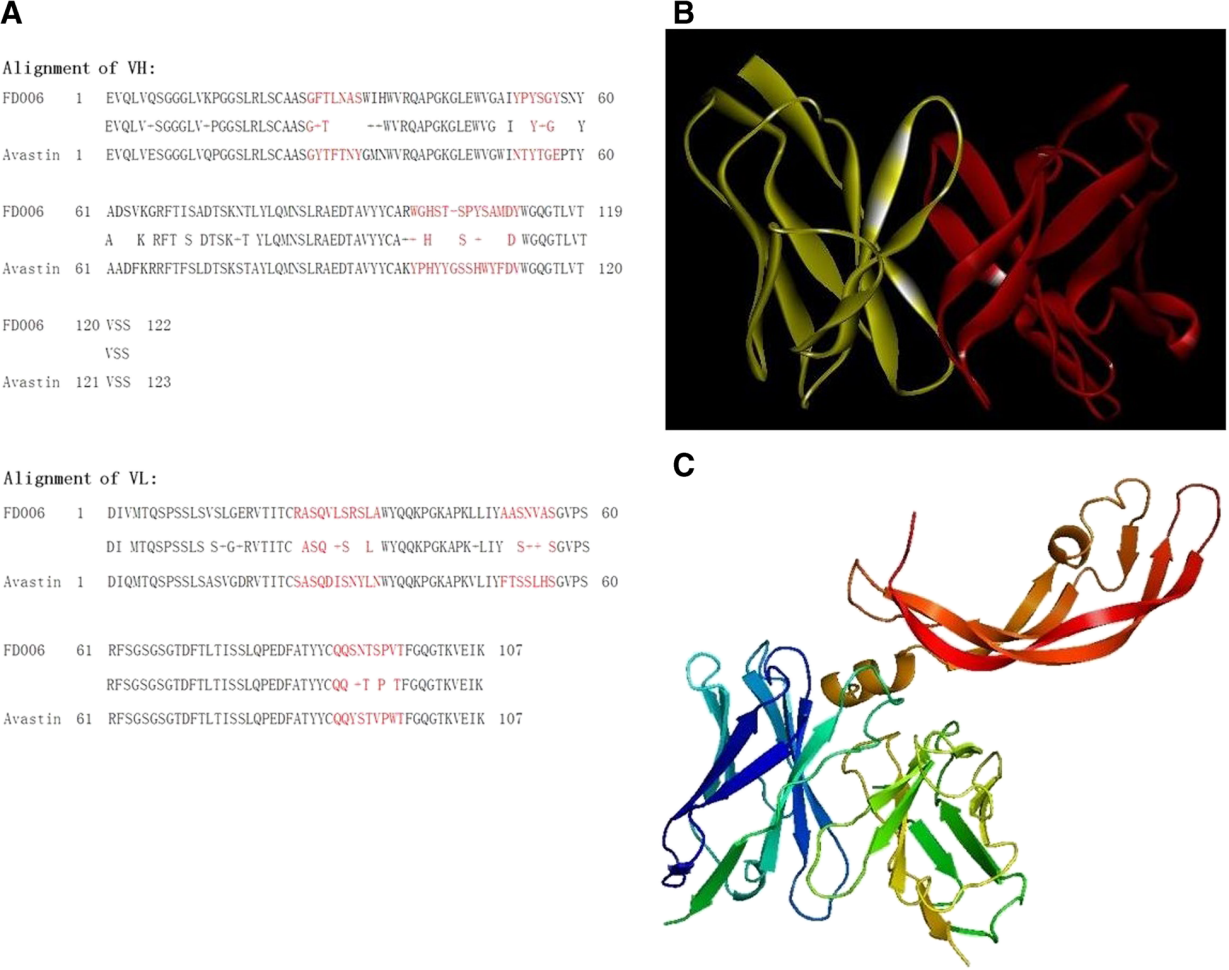
**Theoretical analysis of FD006 to bind VEGF. A**: The amino acid residues and CDR region classification of FD006 (versus Avastin/bevacizumab); **B**: The 3-D structure of variable region in FD006 using computer-guided homology modeling and molecular dynamics methods. The yellow ribbon denoted the light chain variable region and the red ribbon denoted the heavy chain variable region; **C**: The 3-D complex structure of FD006 variable region and VEGFA obtained from computer-guided molecular docking and dynamics methods. The upper red ribbon denoted VEGF, the lower left (blue) ribbon was FD006-VL and the lower right (green and yellow) denoted FD006-VH.

Using antibody phage display library, we obtained a novel anti-VEGFA antibody, named as FD006. After sequencing, the amino acid residues of the variable region in FD006 were determined as shown in Figure 1A. Using computer-guided homology modeling method, the Stable 3-D structure of FD006 variable region was constructed and shown in Figure 1B. Based on the crystal structure of VEGFA, the complex structure of VEGFA and FD006 was constructed and optimized (Figure 1C) using computer-guided molecular docking method. Using interactive graphics and the hard-sphere approximation for atoms, we found that the FD006 variable region was fitted into the binding site of VEGFA which identified by bevacizumab. The binding mode of FD006, *e.g.* binding energy and epitope, was analyzed and shown in Table 1, which showed that the binding mode between FD006 and VEGF was similar to bevacizumab and VEGF.

**Table 1:** The predicted binding energy (kCal/mol) and epitope between antibody (FD006 or bevacizumab) and antigen (VEGFA)

	**Binding energy (kcal/mol)**	**Identified epitope**
FD006-VEGF	-168.24	F^17^M^18^D^19^, Y^21^Q^22^R^23^, Y^25^, L^66^E^67^, N^100^K^101^
bevacizumab-VEGF	-156.35	F^17^M^18^D^19^, Y^21^Q^22^R^23^, Y^25^, K^101^

### FD006 possessed similar or even higher affinity than bevacizumab

**Figure 2:**
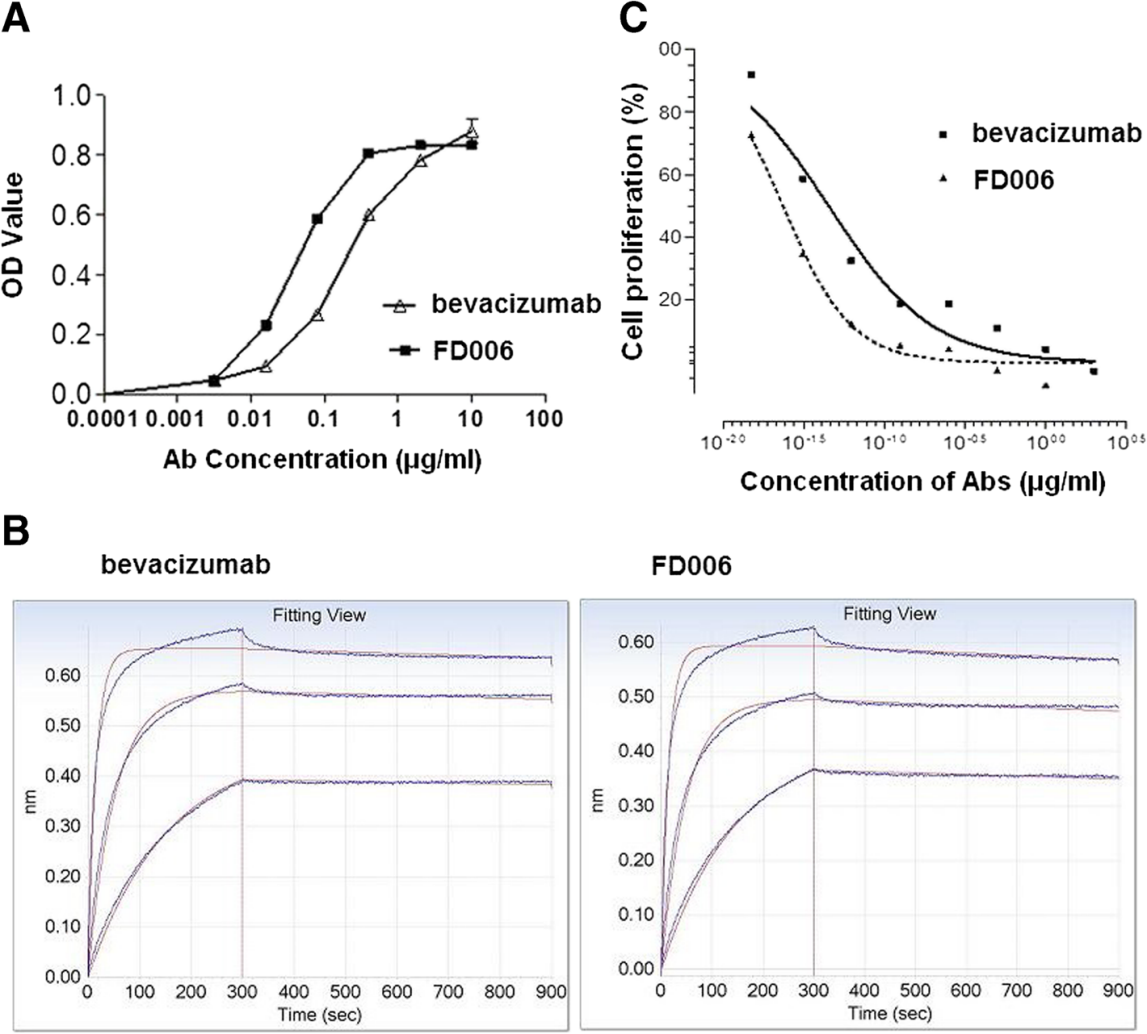
**Antigen binding identification of FD006 to bind VEGF. (A)** The binding activity of FD006 by ELISA; **(B)** Affinity detection of FD006 by binding kinetics assays; **(C)** The inhibitory effects of FD006 on VEGF-induced human umbilical vein endothelial cell (HUVEC) proliferation. The proliferation percentage was calculated using the formula OD_sample_/OD_untreated_ × 100 %. Data points represent the mean 6 SD of values acquired in duplicate.

ELISA assay was performed to determine the antigen-binding specificity of FD006. As shown in Figure 2A, it was shown that both bevacizumab and FD006 could bind to VEGF on a dose-dependent manner. In addition, the EC50 of FD006 was about 0.037 μg/mL while that of bevacizumab was 0.18 μg/mL, suggesting that FD006 might possess stronger binding ability than bevacizumab (5-fold). Further experiments indicated the interacting kinetics of two mAbs to bind to VEGF (Figure 2B), displaying approximately 2-fold higher affinity of FD006 than bevacizumab (Table 2) due to its slower dissociation rate; besides, the inhibitory ability of FD006 against the VEGF-induced cell proliferation in HUVEC was identified. The IC50 value of FD006 was approximately 0.031 ± 0.0064 μg/ml, indicating its somewhat better antiangiogenic activity of FD006 than bevacizumab (0.047 ± 0.0081 μg/ml) (Figure 2C).

**Table 2:** Interacting kinetics of bevacizumab and FD006 binding to VEGF

	**KD (M)**	**Kon (1/Ms)**	**Kdis (1/s)**	**Full R2**
bevacizumab	6.50E-10	1.75E + 05	1.02E-04	0.992536
FD006	3.11E-10	1.48E + 05	4.59E-05	0.992964

### FD006 inhibited in vivo neovascularization in CoNV

**Figure 3:**
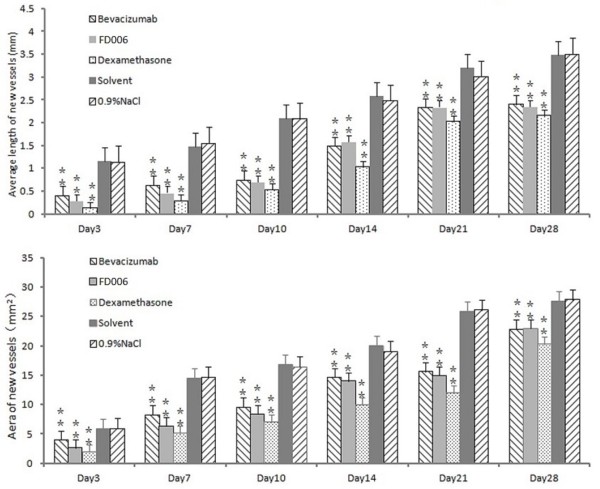
**The mean length and area of the corneal neovascularization.** 0.9% NaCl treated group and solvent group were set as controls. Results are represented as mean ± SEM. *p < 0.05, **p < 0.01.

**Figure 4:**
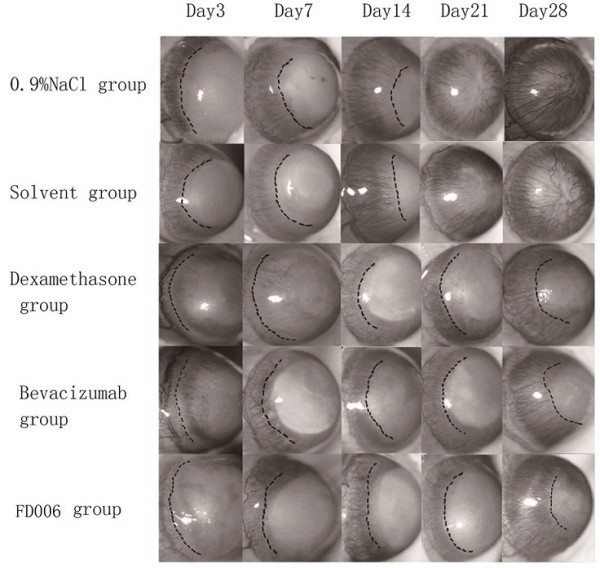
**Typical images of rat corneal neovascularization caused by alkali burn.** The dotted lines represent the margin of the corneal neovascularization.

**Figure 5:**
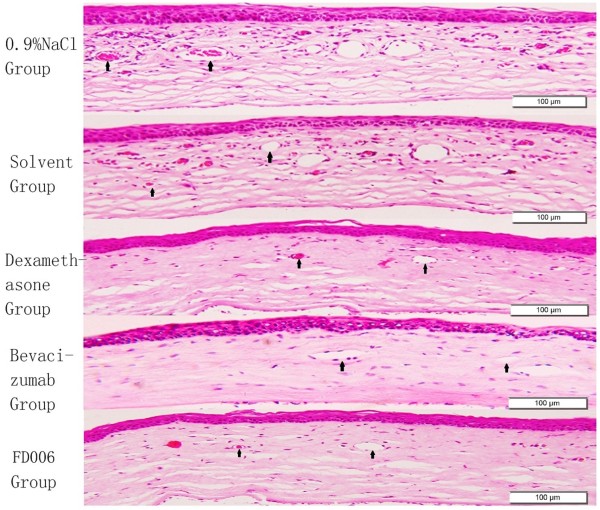
**Hematoxylin and eosin (HE) staining observation of corneas.** At day 7, corneal neovascularization of various sizes (black arrowhead) were present in the superficial central corneal stroma after alkali burn (×200) and neovascularization containing RBCs in the corneal stroma were indicated with black arrowheads. Cornea stroma exhibited edema and mononuclear inflammatory response. In this plane of section, the corneal epithelium is relatively normal. Bar = 100 μm.

Alkali-induced CoNV model is a simple model system for the study of neovascularization. After the alkali-soaked filter paper was removed from the cornea, the central cornea appeared opaque with a clear margin. After modeling, vessels began sprouting into the cornea from the limbus. It was demonstrated that the subconjunctival administration of FD006, bevacizumab and dexamethasone could all significantly inhibit CoNV in NaOH cauterized rats compared with the control group (p < 0.01) (Figures 3 and 4); furthermore, compared with bevacizumab, FD006 was a little more effective on inhibiting CoNV to reduce the length and area of the corneal neovasculature at day 3 and day 7 (0.01 < P < 0.05). However, since day 14, there was no obvious difference between the two groups (p > 0.05). Meanwhile, the two antibodies were less effective than dexamethasone (p < 0.01). Typical HE staining images of the rats’ CoNV were shown in Figure 4, which also displayed weaker vascularization in the FD006 group, bevacizumab group and dexamethasone group contrasting to that in the control group (Figure 5).

### FD006 downregulated the expression of neovascularization-related molecules in the cornea

**Figure 6:**
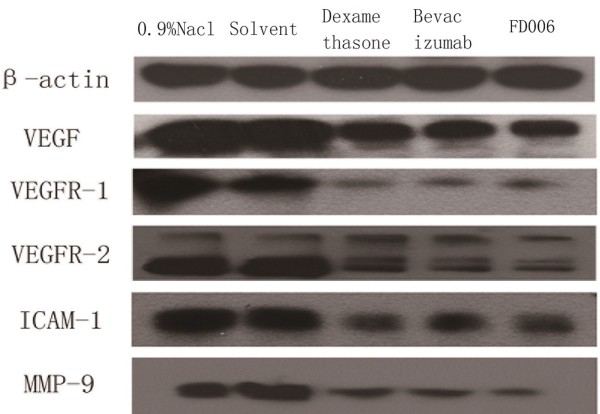
**The protein expression of neovascularization-related signal molecules in the cornea.** β-actin served as an internal standard. The protein expression of VEGF, MMP-9, VEGFR-1, VEGFR-2 and ICAM-1 in the cornea was significantly weaker in antibody- or dexamethasone- treated samples.

According to western blot analysis, the subconjunctival administration of FD006 or bevacizumab significantly decreased the protein expression of VEGF, VEGFR-1, VEGFR-2, MMP-9 and ICAM-1, while 0.9% NaCl treated group and solvent group displayed visibly high expression of the above factors in the cornea (Figure 6). It might be the possible mechanism of FD006’s inhibitory ability against neovascularization *in vivo*, indicating that FD006 might be a favorable anti-angiogenic candidate drug.

## Discussion

The normal cornea is an avascular structure. The formation of neovascularization may be a physiological response to various stimuli; however, the chronic and persistent upregulation of pro-angiogenic factors may result in pathological CoNV, which can lead to blindness. For it was reported that VEGF is required for inflammatory neovascularization and a crucial endogenous corneal angiogenic factor, VEGF was confirmed as the key target for blocking the cascade of neovascular formation [16,17]. VEGF is a vascular endothelial cell mitogen and one of the key regulators of angiogenesis in various physiological and pathological conditions [14]. By binding to VEGFR, VEGF triggers its tyrosine phosphorylation and induces a signaling cascade that promotes growth, survival, migration, differentiation of endothelia cells as well as mobilizing endothelial progenitor cells from the bone marrow into the peripheral circulation [16-18]. The angiogenic activity of VEGF is believed to be regulated by two high affinity receptor tyrosine kinases, VEGFR-1 (fms-like tyrosine kinase, Flt-1) and VEGFR-2 (kinase insert domain receptor, Flk-1), which is reported to be the dominant angiogenic activity receptor of VEGF. Although the complications of dexamethasone such as cataract and secondary glaucoma have limited its clinical use, it is still a powerful anti-VEGF agent; bevacizumab is also considered as an effective anti-VEGF agent that can bind to soluble VEGF, preventing receptor binding [19,20].

We discovered that the protein expression of ICAM-1 and MMP-9 was significantly higher in the control groups than in the other groups. FD006 reduced the expression of ICAM-1 and MMP-9 compared with the control groups (Figure 6). ICAM-1 (Intercellular adhesion molecule 1) is one of cell surface glycoproteins that are typically expressed on endothelial cells. ICAMs may play a critical role in regulating leukocyte migration and accumulation at sites of inflammation, thus generating inflammatory response and damage to tissue [21]. Corneal fibroblasts expressed ICAM-1 and VCAM-1 when activated with IL-4 and TNF-alpha [22]. Luo *et al.* discovered that VEGF and b-FGF can facilitate the expression of ICAM-1 [23]. Therefore we inferred that the expression level of ICAM-1 was lower in FD006 group because there may be a feedback reaction where the decreased VEGF expression influences the release of ICAM-1 after FD006 blocked the VEGF signaling.

MMPs play indispensable roles in the formation of CoNV and are one of the most potent proangiogenic factors. MMP-9, also known as gelatin B, plays an important role in degrading the ECM and basement membrane during organizational restructuring and angiogenesis. MMPs can promote the migration of endothelial cells by destroying connections between the cells and the extracellular matrix [24]. Particularly, the synergistic actions of MMPs and VEGF have been discovered in angiogenesis, and studies have already demonstrated that VEGF and MMPs influence each other during angiogenesis. MMP can enhance VEGF release and modulate VEGF expression [25-28]. Additionally, VEGF increases the release of the MMPs and decreases the release of the tissue inhibitor of metalloproteinase, whereas MMPs activate the angiogenic activity of VEGF. We believe that, among ICAM-1, MMP-9 and VEGF, there is a cascade of chain reactions that influence each other. However, our hypotheses require further investigation.

In this study, we screened and predicted the novel anti-VEGF monoclonal antibody FD006 to have similar affinity to bevacizumab (Figure 1 and Table 1). Further experiments testified that both bevacizumab and FD006 could bind to VEGF specifically on a dose-dependent manner; meanwhile, FD006 showed somewhat stronger affinity to bind VEGF than bevacizumab by both ELISA (5-fold) and binding kinetics assays (2-fold) mainly because of its slower dissociation rate (Table 2). In principle, the higher affinity of anti-VEGF means higher efficiency to neutralize VEGF; furthermore, FD006 seemed to have a better inhibitory effect on the VEGF-induced proliferation of HUVEC than bevacizumab, which was consistent with antigen binding assays (Figure 2).

The alkali burn-induced CoNV model has been widely used to investigate the mechanism of corneal neovascular formation. Here, this method also induced CoNV successfully. Several studies reported that bevacizumab was more effective via subconjunctival administration for CoNV compared with via topical eye drops [19,20], therefore in this study FD006 was subconjunctivally injected in alkali-burn induced corneal neovascularizaiton to evaluate its biological properties *in vivo*. It was demonstrated that FD006 could inhibit corneal neovascular formation. Particularly, during the early stage after alkali burn, it seemed a little better than bevacizumab (Figures 3, 4 and 5). The reason might be the higher ability of FD006 to bind VEGF. However, at the late stages, FD006 and bevacizumab had an equal therapeutic effect on inhibiting CoNV. Besides, FD006 significantly decreased the expression of VEGF, VEGFR-1, VEGFR-2, ICAM-1, and MMP-9 in cornea compared with the control group (Figure 6), explaining partially why FD006 had satisfactory anti-neovascularization effect.

## Conclusion

This study displayed the pharmacological characteristics of FD006, which were similar or a little superior to bevacizumab, such as higher affinity, better anti-neovascularization function *in vitro* and *in vivo*, indicating that FD006 might be a promising agent in the treatment of human CoNV in the future.

## Methods

### Chemicals and antibodies

FD006 is a potent recombinant humanized monoclonal antibody directed against VEGF and prepared in our lab. The commercial anti-VEGF antibody bevacizumab (Avastin, 100 mg/4 ml) was purchased from Roche Pharmaceutical (Genentech, South San Francisco, USA), analyzed in our theoretical platform and set as positive control. Bevacizumab was dialyzed with the same solvent as FD006 in this study.

### Computational modeling and docking

Based on the variable heavy (VH) and light chain (VL) sequences of FD006, sequence alignmment were performed using BLASTp (http://www.ncbi.nlm.nih.gov) software with PDB database. And then, the 3-D structure of the antibody FD006 variable region was constructed using computer-guided homology modeling method based on the crystal structures of VH domain of B20-4 Fab (PDB code: 2FJH) and VL domain of B20-4 Fab (PDB code: 2FJH). After constructing the backbone of the Fv fragment, the side chains were replaced using rotamer libraries and algorithm implemented in the Homology package of InsightII 2000 (MSI, San Diego, CA). Furthermore, the structure of the FD006 variable region was optimized under CVFF force field [29] using molecular dynamics method. On the basis of the crystal structure of the extracellular domain of VEGFA (PDB code: 2FJH [30], the partial charges of the VEGFA and FD006 Fv fragment were assigned. According to the crystal complex structure of VEGFA and its functional antibody bevacizumab (PDB code: 2FJH), the FD006 Fv fragment was docked manually into the binding site of VEGFA. The antigen-antibody complex was relaxed by energy minimization and subsequent molecular dynamic simulations in vacuo using the CVFF forcefield. Molecular dynamic simulations were performed using the NVT ensemble of the Discover 3.0 package (MSI). The binding energy and identified domain between FD006 and VEGFA were analyzed with distance geometry and computer graphics technology.

### ELISA analysis

ELISA plates were coated with 100 μl/well 0.5 μg/ml VEGF. Bevacizumab or FD006 were diluted (10, 2, 0.4, 0.08, 0.016 and 0.0032 μg/ml) and added for 2 hours’ incubation in 37. After three times of washing, the Peroxidase-conjugated affinipure goat anti-human immunoglobulin G (IgG) was added as the secondary antibody for 45 minutes at room temperature (RT for short, the same below). Binding signals were visualized using o-phenylenediamine dihydrochloride (OPD) substrate and the light absorbance was measured with an ELISA reader at 492 nm.

### Binding kinetics assays

The binding kinetics of bevacizumab or FD006 to VEGF was measured using Bio-Layer Inter-Ferometry on Octet RED (Fortebio, USA). The assay was conducted at 30°C in PBS buffer. Sensor tips were pre-wet for 15 mins in buffer immediately prior to use, and the microplates were filled with 200 μl per well of diluted samples (VEGF) or buffer and agitated at 1000 rpm. The anti-human IgG biosensor were pre-saturated with bevacizumab or FD006 (10 μg/ml) and washed in buffer for 120 seconds, and then transferred to VEGF at concentrations of 10 μg/ml, 3 μg/ml and 1 μg/ml. The VEGF association and dissociation rates were measured for 5mins and 10mins, respectively. The Kinetics parameters (Kon and Koff) and affinities (KD) were calculated from a non-linear global fit using the Octet analysis software. Multiple independent measurements were performed.

### Proliferation assays

Human umbilical vein endothelial cells (HUVECs) (1 × 10^4^ cells/100 μL/well) were seeded in 96-well plates and cultured at 37 for 14 h with Endothelial Cell Medium (ScienCell) supplemented with 5% heat-inactivated FCS, 100 U/ml penicillin, 100U/ml streptomycin, and endothelial cell growth supplement (ScienCell). After low-serum starvation overnight, cells were treated with different concentrations of FD006 or bevacizumab which were pre-incubated with 10 ng/ml VEGF for 30 minutes and incubated at 37, 5% CO2 for 72 hours. Then, 10 μl CCK8 was added to each well and incubated for another 4 hours. The absorbance was measured by spectrophotometer at 450 nm to determine the cell viability.

### Animals

Ninety male Sprague–Dawley rats (6–8 weeks, 180-200 g) were obtained from the Laboratory Animal Centre in Academy of military medical sciences. The right eyes of the animals were used for experiments, and the left eyes were left intact. All rats were carefully examined before the procedure using a slit lamp microscope and the corneas with any defects, new vessels, or cataract were excluded.

The animal studies were approved by Animal Ethics Committee of Institute of Basic Medical Sciences. All procedures involving animal eye studies were conducted strictly in accordance with the ARVO Statement for the Use of Animals in Ophthalmic and Vision Research.

### Alkali burn model

Prior to the procedure, the rats were anesthetized using an intraperitoneal injection of 3.5 ml/kg 10% chloral hydrate (General Hospital of PLA, Beijing), and the eyes were treated with a topical application of 0.5% proparacaine hydrochloride (Alcain; Alcon, USA). Then, a 3 mm diameter Whatman 3# filter paper was soaked in 5 μL of 1 M sodium hydroxide for 60 s and then placed in the center of the cornea for 40 s. The burned area and conjunctival sac was then irrigated with 60 ml saline for 1 minute.

### Animal treatment

After modeling, ninety rats were randomly divided into five groups (eighteen rats per group) and received a subconjunctival injection with 0.05 ml per rat of (1) 0.9% NaCl, (2) solvent, (3) 0.1% dexamethasone (Dexamethasone sodium phosphate, NCPC.LTD, China), (4) 25 mg/ml bevacizumab and (5) 25 mg/ml FD006 in the superior temporal conjunctiva on the day after modeling. All chemical burns and treatments were performed by one investigator. The operator was blinded to the treatment group from which each cornea was derived. At postoperative days 3, 7, 14, 21 and 28, the eyes were harvested for further studies after the rats were sacrificed with an overdose of 10% chloral hydrate.

### Quantification of CoNV

Slit lamp examinations were performed in a blinded manner on day 3, 7, 14, 21 and 28 by a same operator. The changes in length and area of the corneal neovasculature and related adverse complications were recorded using a Canon Cybershot camera (Canon Corporation, Japan). The corneas were examined and photographed routinely. The photographs were digitized, and the images were analyzed using Image J Software. The length of the CoNV was measured from the edge of the sclera using a reticule on days 3, 7, 14, 21 and 28 after modeling. The area (A) of the CoNV was calculated using the following formula: A = C/12 × π[r^2^ ‒ (r ‒ l)^2^], where C is the clock hours of corneal NV coverage on the cornea, l is the average length of the selected vessels, and r is the radius of the rat cornea.

### Immunohistochemistry

The rat eyeballs were harvested and fixed in 4% paraformaldehyde. The corneas were excised, embedded in paraffin, cut into 5-μm sections and then prepared for hematoxylin and eosin (H&E) staining. The cornea sections were fixed in acetone. The primary antibodies were VEGF-A, fms-like tyrosine kinase (VEGFR-1, Flt-1) and kinase insert domain receptor (VEGFR-2, Flk-1), matrix metalloproteinase-9 (MMP-9) and intercellular adhesion molecule-1 (ICAM-1). Goat anti-mouse IgG was used as the secondary antibody. Then stained samples were observed and photographed using inverted microscopy.

### Western blot

The rats were euthanized, and the corneas were harvested from the treated eyes. The corneas were dissected and frozen at -70°C, then homogenized in ice-cold RIPA lysis buffer solution (Santa cruz, USA). After being centrifuged for 5 minutes at 12,000 revolutions per minute (rpm), the supernatants of the samples were collected. Certain amounts of the protein were separated by electrophoresis on an 8% and 12% sodium dodecyl sulfate-polyacrylamide gel, and transferred to nitrocellulose membranes (Invitrogen, San Diego, CA). Then the membranes were blocked using 5% skim milk in TBST (20 mM Tris, 150 mM NaCl and 0.1% Tween-20) and incubated overnight with primary antibodies: anti-VEGF (Santa Cruz, USA), MMP-9 (Abcam, UK), VEGF receptor 1 (Abcam, UK), ICAM-1 (LifeSpan BioSciences, USA) and VEGF receptor 2 (LifeSpan BioSciences, USA). The secondary polyclonal antibodies were used to detect bound primary antibodies: anti-mouse (EMD Millipore, USA), anti-rabbit (EMD Millipore, USA), and anti-goat (EMD Millipore, USA). After washing, an enhanced chemiluminescence solution (ECL reagent: Santa Cruz, CA) were used to visualize specific lanes.

### Statistical analysis

Statistical analysis was performed using IBM SPSS Version 20.0 software (IBM SPSS, Chicago, IL). Experimental data were analyzed using one-way ANOVA, the Dunnett multiple comparison test, and independent two-sample t-tests. All reported P values are 2-sided and P < 0.05 was considered statistically significant. All results are presented as the mean ± standard error of the mean. All measurements were within 95% confidence limits.

## Competing interests

The authors declare that they have no competing interests.

## Authors’ contributions

QW did in vivo experiments and prepared the manuscript, JY did in vitro binding, cell proliferation experiments and western blot analysis, KT constructed expression vectors, LL selected FD006 from the phage library, LW established the animal model, LT helped do the animal operation, YJ helped observe animals and take photos, JF did theoretical anlaysis, YL purified FD006, BS did antibody preparation in mammalian cells, ML designed the work and did affinity identification, YH designed the work and revised the manuscript. All authors read and approved the final manuscript.

## Authors’ information

Qun Wang and Jing Yang are co-first authors.
